# Progenitor death drives retinal dysplasia and neuronal degeneration in a mouse model of ATRIP-Seckel syndrome

**DOI:** 10.1242/dmm.045807

**Published:** 2020-10-30

**Authors:** Gabriel E. Matos-Rodrigues, Pedro B. Tan, Maurício Rocha-Martins, Clara F. Charlier, Anielle L. Gomes, Felipe Cabral-Miranda, Paulius Grigaravicius, Thomas G. Hofmann, Pierre-Olivier Frappart, Rodrigo A. P. Martins

**Affiliations:** 1 Programa de Biologia Celular e do Desenvolvimento, Instituto de Ciências Biomédicas, Universidade Federal do Rio de Janeiro, Rio de Janeiro, RJ 21941902, Brazil; 2 Max Planck Institute of Molecular Cell Biology and Genetics, Pfotenhauerstraße 108, 01307 Dresden, Germany; 3 Deutsches Krebsforschungszentrum, 69120 Heidelberg, Germany; 4 Institute of Toxicology, University Medical Center of the Johannes Gutenberg University Mainz, Mainz, 55131 Germany

**Keywords:** Apoptosis, DNA damage response, Neurodevelopment, Neurodegeneration, Visual system development, Photoreceptor

## Abstract

Seckel syndrome is a type of microcephalic primordial dwarfism (MPD) that is characterized by growth retardation and neurodevelopmental defects, including reports of retinopathy. Mutations in key mediators of the replication stress response, the mutually dependent partners *ATR* and *ATRIP*, are among the known causes of Seckel syndrome. However, it remains unclear how their deficiency disrupts the development and function of the central nervous system (CNS). Here, we investigated the cellular and molecular consequences of ATRIP deficiency in different cell populations of the developing murine neural retina. We discovered that conditional inactivation of *Atrip* in photoreceptor neurons did not affect their survival or function. In contrast, *Atrip* deficiency in retinal progenitor cells (RPCs) led to severe lamination defects followed by secondary photoreceptor degeneration and loss of vision. Furthermore, we showed that RPCs lacking functional ATRIP exhibited higher levels of replicative stress and accumulated endogenous DNA damage that was accompanied by stabilization of TRP53. Notably, inactivation of *Trp53* prevented apoptosis of *Atrip*-deficient progenitor cells and was sufficient to rescue retinal dysplasia, neurodegeneration and loss of vision. Together, these results reveal an essential role of ATRIP-mediated replication stress response in CNS development and suggest that the TRP53-mediated apoptosis of progenitor cells might contribute to retinal malformations in Seckel syndrome and other MPD disorders.

This article has an associated First Person interview with the first author of the paper.

## Abstract (precis)

**Summary:** Retinopathies have been reported in primordial dwarfism syndromes. We show that the loss of *Atrip*, a gene mutated in Seckel syndrome, causes photoreceptor degeneration owing to p53-dependent apoptosis of retinal progenitors during development.

## INTRODUCTION

Maintenance of genomic stability is crucial for human health, particularly to prevent neurologic diseases (McKinnon, 2017). Several inherited syndromes that affect the development of the central nervous system (CNS) are caused by mutations in DNA damage response (DDR) genes (Jackson and Bartek, 2009). The ATRIP-ATR signalling complex is a central mediator of the replicative stress response (RSR) (Saldivar et al., 2017), and mutations in both genes have been reported in Seckel syndrome (Mokrani-Benhelli et al., 2013; O'Driscoll et al., 2003; Ogi et al., 2012). Seckel syndrome belongs to a group of microcephalic primordial dwarfism syndromes (MPDs) that includes the osteodysplastic MPD types I/III and II, Silver–Russell and Meier–Gorlin syndromes (Bober and Jackson, 2017; Khetarpal et al., 2016). Seckel syndrome and other MPDs are characterized by neurodevelopmental defects including microcephaly and microphthalmia. Although not a hallmark of all MPDs, there are various reports of ocular abnormalities and severe functional deficits, such as loss of vision, especially for MPDII and Seckel syndrome (Chen et al., 2019; Erdöl et al., 2003; Guirgis et al., 2001; Hall et al., 2004; Krzyżanowska-Berkowska et al., 2014; Lim and Wong, 1973; Robbin, 1985; Shanske et al., 1997; Vakili, 2019). Genetic and functional studies have identified that Seckel mutants of *ATRIP* and *ATR* impair the RSR (Murga et al., 2009; O'Driscoll et al., 2003; Ogi et al., 2012). However, it is not completely understood how defective RSR during development leads to CNS malformations.

Studies using previous mouse models of Seckel syndrome endeavored to explain how defective RSR leads to CNS malformations. Analysis of mice harboring a humanized Seckel mutation of *Atr* revealed apoptosis of neural progenitor cells and transformation related protein 53 (TRP53) accumulation in embryonic brains (Murga et al., 2009). CNS-specific inactivation of *Atr* also induced DNA damage and TRP53-mediated apoptosis of cortical and cerebellar neural progenitor cells. Notably, disruption of apoptotic pathways (e.g. *Trp53* inactivation) did not rescue the growth impairment and neuropathology of these brain tissues (Lang et al., 2016; Lee et al., 2012). Therefore, it remains to be determined which cellular events triggered by defective RSR cause the neurodevelopmental malformations that lead to functional deficits in the adult CNS.

Electroretinogram analysis showed the lack of photoreceptor responses in a genetically undiagnosed Seckel syndrome and other MPD patients (Guirgis et al., 2001; Martin et al., 2014). These findings suggest that retina-specific defects might contribute to visual impairments. The retina is the neural part of the eye responsible for the detection and preprocessing of the visual stimuli before transmission to the visual centers of the brain (Dowling, 1987). The mature retina of vertebrates is organized in functional layers, and the primary sensory neurons, photoreceptors, occupy the apical-most layer of the retina, known as the outer nuclear layer (ONL) (Hinds and Hinds, 1979; Rich et al., 1997). Different types of retinal interneurons responsible for processing and relay of the visual information are located within the inner nuclear layer (Diamond, 2017; Stell, 1972). The basal layer is composed mainly of retinal ganglion cells, which are projection neurons that connect the retina to the brain (Hinds and Hinds, 1974; Sernagor et al., 2001). To generate such a complex laminated structure, progenitor cell proliferation and neurogenesis need to be coordinated precisely with the migration of newborn neurons to appropriate layers (Agathocleous and Harris, 2009; Amini et al., 2017). The final laminar configuration is crucial for the proper assembly of the retinal circuitry, and defects in these cellular processes can lead to visual impairment (Amini et al., 2017; Hoon et al., 2014).

Although retinal malformations have been found in case reports of Seckel syndrome patients, the molecular and cellular mechanisms that impair retinogenesis are still unclear. To elucidate whether defective RSR drives retinal malformations, we conditionally inactivated *Atrip* in retinal progenitor cells (RPCs) and in postmitotic retinal neurons. Loss of ATRIP in photoreceptor neurons did not affect their survival or function. In contrast, RPC-specific inactivation of *Atrip* led to apoptosis of progenitor cells, defective retinal lamination, extensive neurodegeneration and loss of visual acuity. Remarkably, inactivation of *Trp53* in the *Atrip*-deficient retinas rescued the apoptosis of RPCs and completely restored retinal structure and function. These data support a crucial contribution of the ATRIP-mediated RSR to retinal development and provide new insights into the mechanisms underlying visual impairments following deregulated RSR during retinogenesis.

## RESULTS

### *Atrip* inactivation in the retina disrupts lamination and leads to photoreceptor degeneration and blindness

**Fig. 1.**
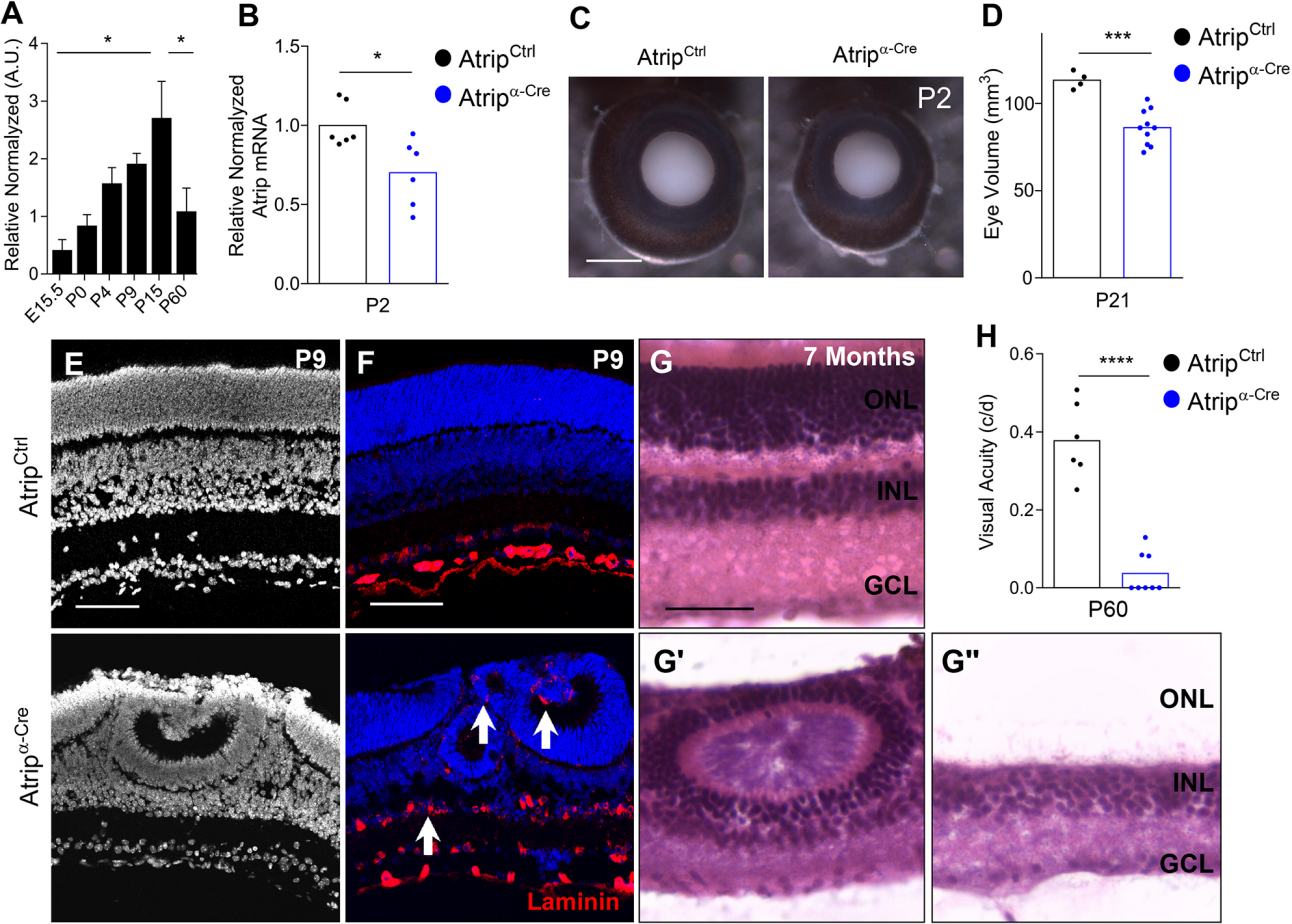
**Retinal progenitor cell-specific inactivation of *Atrip* leads to lamination defects and neurodegeneration.** (A) Real-time RT-PCR for *Atrip* in the wild-type mouse retinas at E15.5, P0, P4, P9, P15 and P60 (*n*=3). (B) Real-time RT-PCR for *Atrip* in *Atrip^Ctrl^* and *Atrip^α-Cre^* retinas at P2. (C) Representative images of the *Atrip^Ctrl^* and *Atrip^α-Cre^* eyes at P2. (D) Measurements of eye volume for *Atrip^Ctrl^* and *Atrip^α-Cre^* at P21. (E) DAPI staining of *Atrip^Ctrl^* and *Atrip^α-Cre^* retinal sections at P9. (F) Immunofluorescence for laminin in *Atrip^Ctrl^* and *Atrip^α-Cre^* retinas at P9. White arrows indicate misplaced laminin staining in *Atrip^α-Cre^* retinas. (G) Hematoxylin and Eosin staining in *Atrip^Ctrl^* and *Atrip^α-Cre^* retinal sections at 7 months. (H) Behavioral optomotor response analysis in the *Atrip^Ctrl^* and *Atrip^α-Cre^* mice at P60. Statistical analysis: one-way ANOVA followed by Tukey's post hoc test (A); unpaired Student's *t*-test (B,D,H). *P<0.05; ***P<0.001; ****P<0.0001. Error bars in A indicate s.e.m. Scale bars: 1 mm in C; 100 µm in E, F and G. c/d, cycles/degree; GCL, ganglion cell layer; INL, inner nuclear layer; ONL, outer nuclear layer.

Mutations in *ATR* and *ATRIP* are among the known causes of Seckel syndrome (O'Driscoll et al., 2003; Ogi et al., 2012), and ocular and retinal anomalies have been reported (Aktas et al., 2013; Erdöl et al., 2003; Guirgis et al., 2001; Robbin, 1985). However, the cellular mechanisms by which loss of ATR-ATRIP function causes retinal malformations still await exploration. To shed light on this question, we investigated how the retina develops after inactivation of *Atrip*. Gene expression analysis by real-time RT-PCR in the mouse retina revealed the presence of both *Atrip* and *Atr* transcripts in the embryonic and postnatal stages (Fig. 1A; Fig. S1A). Higher levels of ATR protein were observed in earlier proliferative stages of retinal development (Fig. S1B), suggesting that the ATRIP-ATR complex might play a role in RPCs throughout mouse retinogenesis.

A compound heterozygous mutation of *ATRIP* previously described in a Seckel syndrome patient was shown to reduce ATRIP protein levels and to impair RSR in cells (Ogi et al., 2012). To investigate ATRIP function in retinal development, we generated an *Atrip^α-Cre^* mouse (*Atrip^F/F^;* *α-Cre*^+/−^), in which a *Pax6-*based transgene drives Cre expression in the RPCs of the retinal periphery around embryonic day (E) 11 (Marquardt et al., 2001). Consistent with the spatial pattern of *α-Cre*-mediated recombination, RT-PCR analysis revealed a ∼30% reduction in *Atrip* mRNA content in *Atrip^α-Cre^* retinas at postnatal day (P) 2 (Fig. 1B). Inactivation of *Atrip* specifically in the RPCs impaired eye growth (Fig. 1C) and led to a mild microphthalmia (Fig. 1D). Characterization of the retinal structure showed severe disorganization of retinal lamination in *Atrip^α-Cre^* retinas in the postnatal stages (Fig. 1E). *Atrip^α-Cre^* retinas were marked by aberrant retinal lamination, including rosette formation and ectopic deposits of a component of the basal lamina (laminin) (Fig. 1F). Following the lamination defects, we observed extensive neuronal degeneration in *Atrip^α-Cre^* retinas, particularly of photoreceptor neurons, leading to complete elimination of the ONL in the periphery of the retina (Fig. 1G). In accordance, analysis of the optomotor response in a virtual-reality test revealed severe visual acuity impairment (Fig. 1H). These results demonstrated that the ATRIP-ATR complex is essential for the maintenance of retinal integrity and, therefore, visual function.

**Fig. 2.**
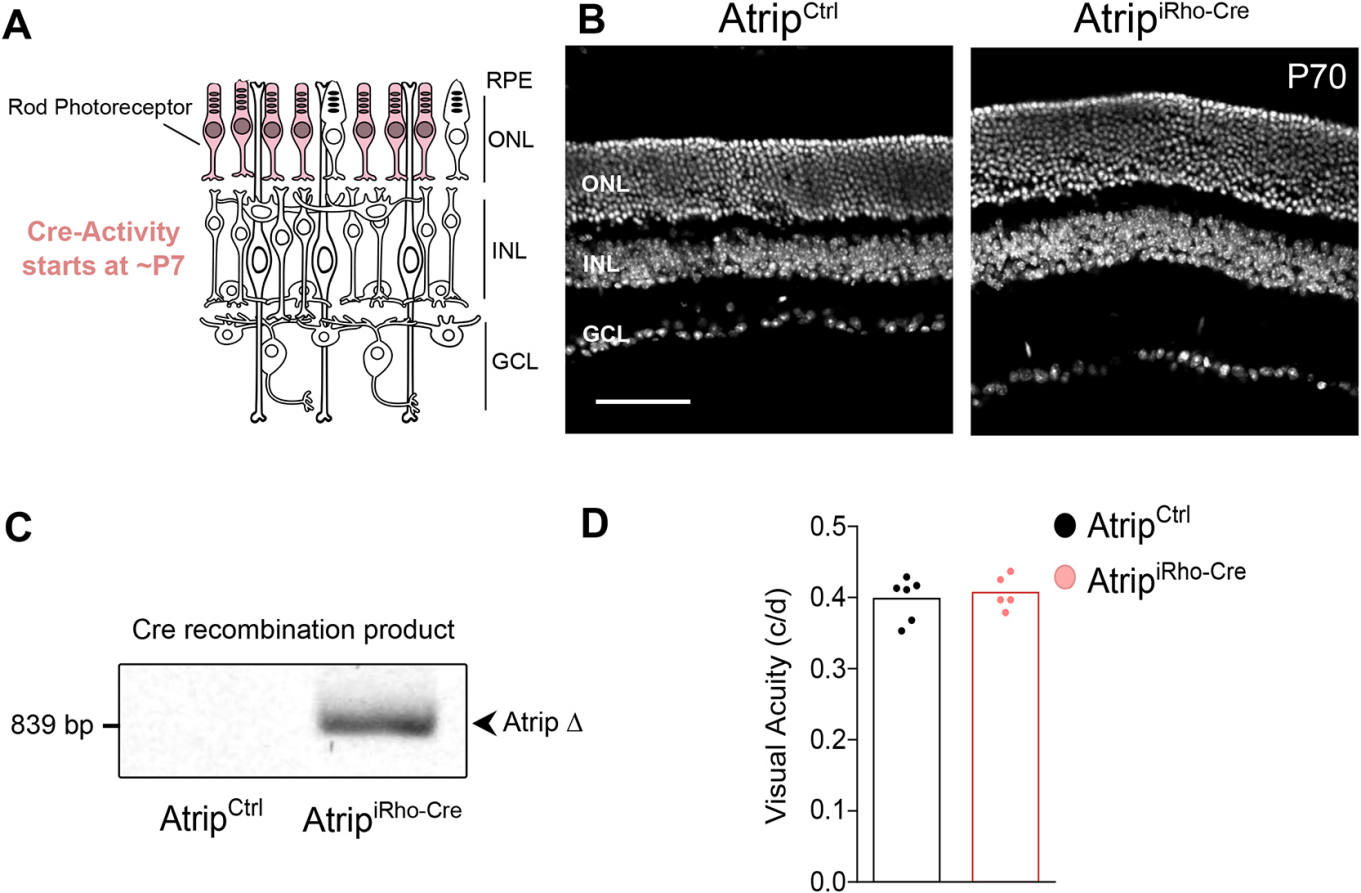
**Conditional inactivation of *Atrip* in rod photoreceptors does not lead to photoreceptor loss or** **impairment of vision.** (A) Schematic representation of the rod photoreceptor-specific *Atrip* inactivation by iRho-Cre. (B) Representative confocal images of DAPI staining of the P70 retinal sections of *Atrip^Ctrl^* and *Atrip^iRho-Cre^* (rod photoreceptor-specific inactivation of *Atrip*). (C) PCR analysis of the iRho-Cre-mediated recombination of the *Atrip* flox transgene in *Atrip^Ctrl^* and *Atrip^iRho-Cre^* retinas at P70. (D) Behavioral optomotor response analysis in 7-month-old *Atrip^Ctrl^* and *Atrip^iRho-Cre^* mice. Scale bar: 100 µm. c/d, cycles/degree; GCL, ganglion cell layer; INL, inner nuclear layer; ONL, outer nuclear layer; RPE, retinal pigmented epithelium.

In a mouse model of ATR-Seckel, it was proposed that photoreceptor degeneration was caused by a role of ATR in the connecting cilia of photoreceptor neurons (Valdés-Sánchez et al., 2013). Thus, we tested whether ATRIP is required for photoreceptor neuron homeostasis by inactivating *Atrip* exclusively in the postmitotic rod photoreceptors using *iRho-Cre* (Li et al., 2005) (Fig. 2A). Interestingly, adult retinas of *Atrip^iRho-Cre^* (*Atrip^F/F^; iRho-Cre*^+/−^) mice displayed normal lamination and ONL (Fig. 2B). As previously reported (Li et al., 2005), a PCR assay confirmed Cre recombinase activity in *Atrip^iRho-Cre^* retinas (Fig. 2C). In contrast to the *Atrip^α-Cre^* mice, the *Atrip^iRho-Cre^* mice exhibited normal visual acuity at 7 months of age (Fig. 2D). These data indicate that ATRIP is not required for the survival or function of postmitotic photoreceptors and suggest that photoreceptor degeneration in *Atrip^α-Cre^* retinas is likely to be caused by defects in the progenitor cells.

### Cell proliferation and neurogenesis are not severely affected in *Atrip*-deficient retinas

**Fig. 3.**
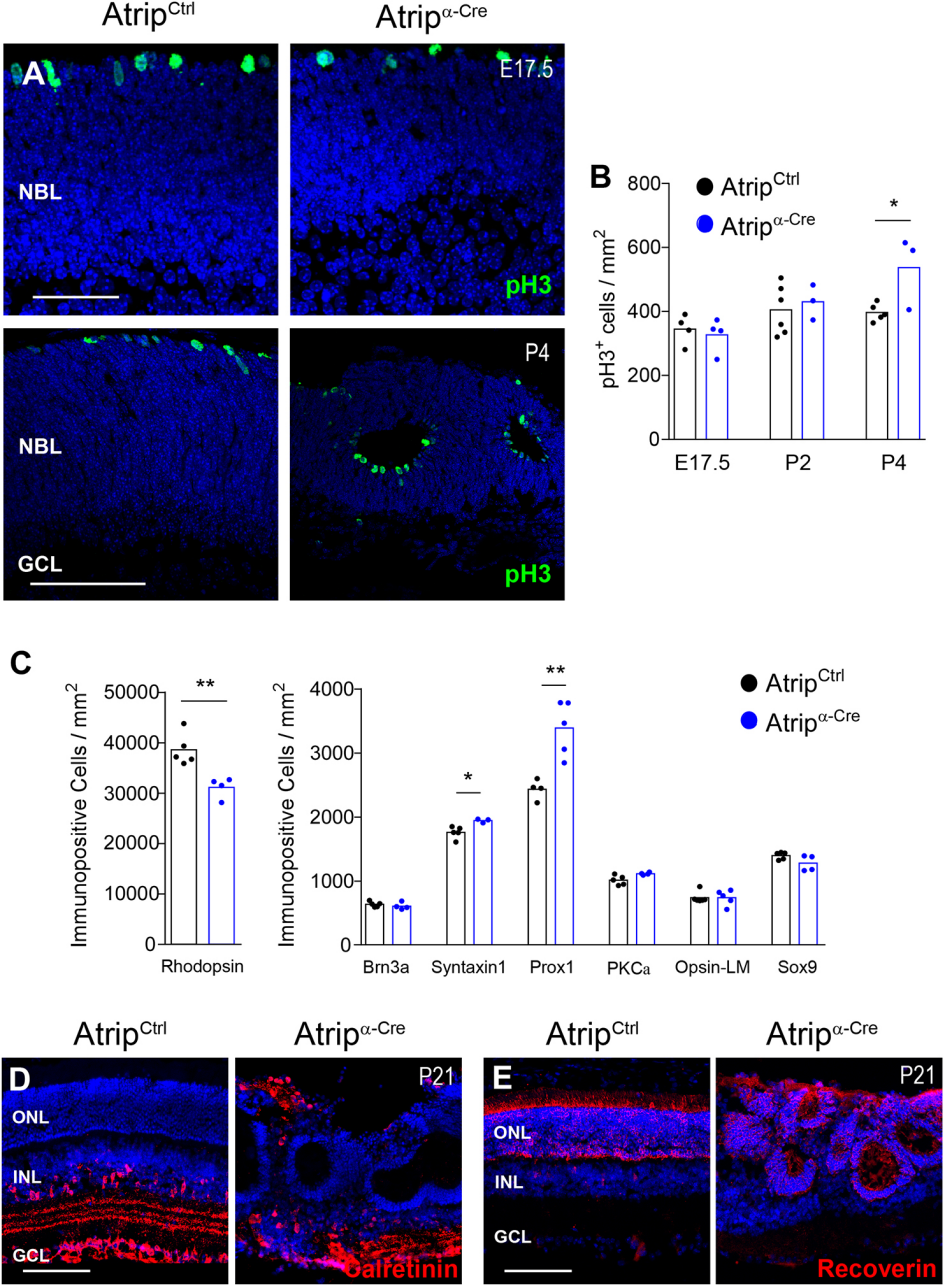
***Atrip* inactivation in RPCs mildly affects retinal neurogenesis.** (A,B) Representative images of pH3 immunostaining (A) and quantification of the pH3^+^ cells (B) in the *Atrip^Ctrl^* and *Atrip^α-Cre^* retinas at E17.5, P2 and P4. (C) Quantification of cell type-specific markers in the *Atrip^Ctrl^* and *Atrip^α-Cre^* retinas at P14. Rhodopsin^+^ (rod photoreceptors), Brn3a^+^ (ganglion cells), syntaxin^+^ (amacrine cells), Prox1^+^ (horizontal and amacrine cells), PKCα^+^ (bipolar cells), Opsin-LM^+^ (cone photoreceptors) and Sox9^+^ (Müller glia) cells were quantified. (D,E) Calretinin (D) and recoverin (E) immunostaining in *Atrip^Ctrl^* and *Atrip^α-Cre^* retinas at P21. Statistical analysis: Student's *t*-test. *P<0.05; **P<0.01. Scale bars: 50 µm at E17.5 in A; 100 µm at P4 in A, and in D and E. GCL, ganglion cell layer; INL, inner nuclear layer; NBL, neuroblastic layer; ONL, outer nuclear layer.

To gain a better understanding of the cellular basis of the compromised morphogenesis of *Atrip^α-Cre^* retina, we evaluated the impact of *Atrip* inactivation on cell proliferation and retinal neurogenesis. Initially, we assessed cell proliferation in early embryonic and postnatal stages. No difference in the proportion of mitotic cells was found in the *Atrip^α-Cre^* retinas at E17.5 or in early postnatal stages (P2), as shown by phospho-histone H3 (pH3^+^) staining (Fig. 3A). Consistent with the aberrant lamination, misplaced mitotic cells were observed in the *Atrip*-deficient retinas at P2 and P4 (Fig. 3A; Fig. S2A). Interestingly, a subtle increase in the proportion of mitotic (pH3^+^) (Fig. 3B) and S-phase [bromodeoxyuridine-positive (BrdU^+^)] (Fig. S2B) cells was detected in the *Atrip^α-Cre^* retinas at P4, indicating a mild dysregulation of cell proliferation in the postnatal *Atrip*-deficient retinas.

Dysregulation of cell proliferation can impact neurogenesis in the developing retina (Ma et al., 1998; Zhang et al., 2004). Therefore, we investigated whether the genesis of retinal neurons and/or glia proceeds normally in *Atrip*-deficient retinas. Analysis of the proportion of differentiated retinal cell type markers revealed a slight decrease in the proportion of rhodopsin^+^ cells (rod photoreceptors) and an increase in syntaxin-1^+^ and Prox1^+^ cells (amacrine and horizontal cells). No difference in the proportion of other cell type markers was observed at P14 (Fig. 3C). Consistent with the lamination defects, different neuronal populations had their distribution altered in the adult *Atrip*-deficient retinas. Displaced amacrine cells (calretinin^+^) and rosettes containing recoverin^+^ cells (rod and cone photoreceptors) were observed at P21 (Fig. 3D,E). Given that the alteration in RPC proliferation was exclusively produced in the late postnatal stages and neurogenesis was mildly disturbed, it is likely that additional cellular mechanisms cause the extensive disorganization of the *Atrip*-deficient retinas.

### RPCs lacking ATRIP accumulate DNA damage and undergo apoptosis during embryogenesis

**Fig. 4.**
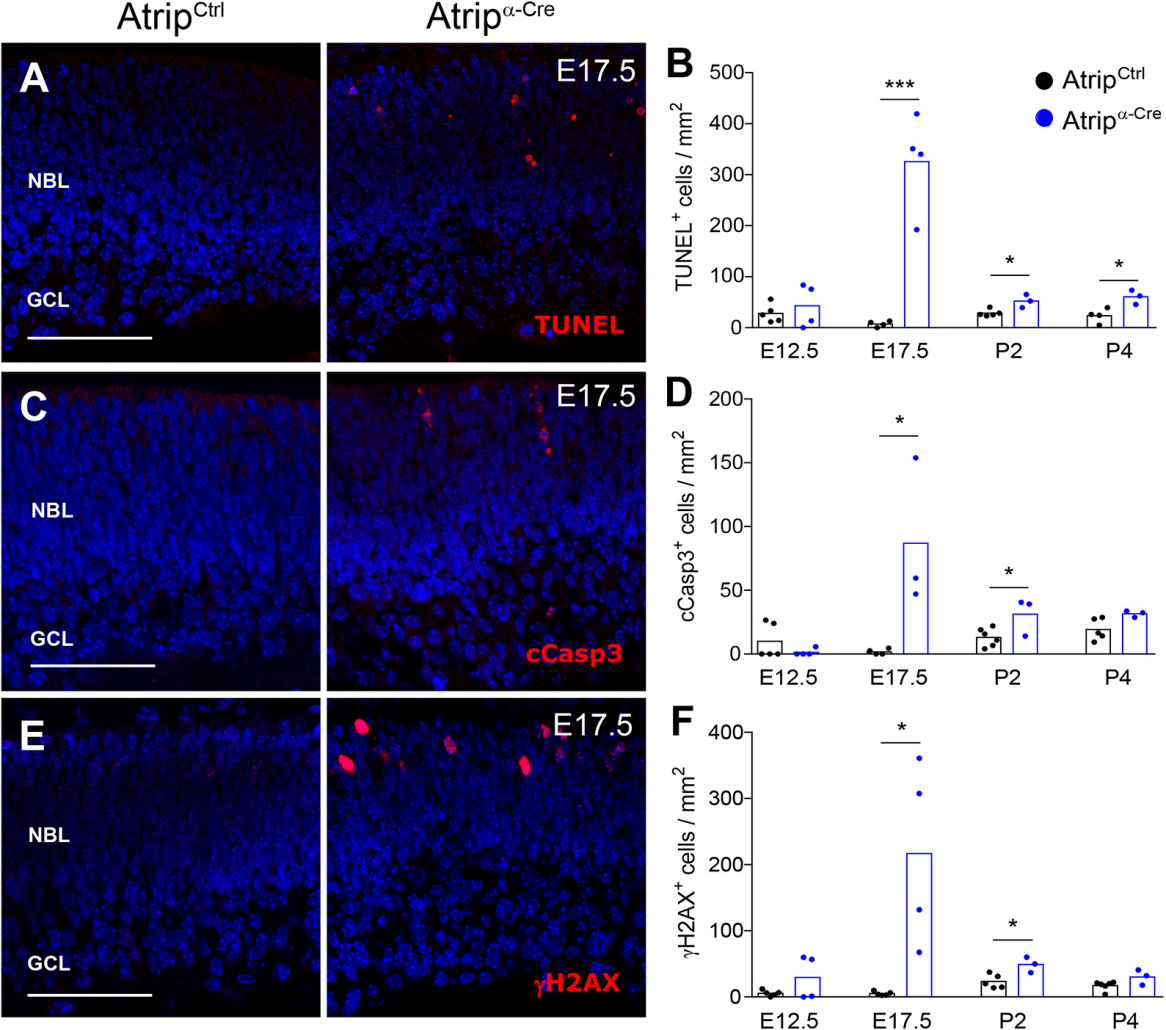
***Atrip* inactivation leads to** **accumulation of DNA damage** **and cell death of RPCs.** (A,C,E) Representative confocal images of TUNEL (A), cCasp3 (cleaved caspase-3) (C) and γH2AX (E) immunostaining in *Atrip^Ctrl^* and *Atrip^α-Cre^* retinas at E17.5. (B,D,F) Quantification of the TUNEL^+^ (B), cCasp3^+^ (D) and γH2AX^+^ (F) cells in the *Atrip^Ctrl^* and *Atrip^α-Cre^* retinas at E12.5, E17.5, P2 and P4. Statistical analysis: unpaired Student's *t*-test. *P<0.05; ***P<0.001. Scale bars: 100 µm. GCL, ganglion cell layer; NBL, neuroblastic layer.

Given that replication-associated DNA damage is frequent during neurogenesis (McKinnon, 2013; Saldivar et al., 2017), we evaluated the impact of ATRIP loss on the accumulation of endogenous DNA damage and cell survival. Shortly after Cre expression (E12.5), no change in apoptosis was detected in the *Atrip^α-Cre^* retinas. In contrast, quantification of TUNEL^+^ and cleaved caspase-3 (cCasp3^+^) cells showed a substantial increase in apoptosis (∼50-fold) in the *Atrip*-deficient retinas at E17.5 and a modest increase in the postnatal stages (P2 and P4) (Fig. 4A-D). Immunostaining for γH2AX, a marker of DNA damage response activation, revealed a coincident temporal pattern of DNA damage accumulation in the embryonic and postnatal RPCs (Fig. 4E,F). In agreement with the pattern of α-Cre-mediated gene inactivation, these phenotypes were observed exclusively in the retinal periphery (Fig. S3). Therefore, similar to *Atr* inactivation in the developing brain (Lang et al., 2016; Lee et al., 2012; Murga et al., 2009), ATRIP loss in the retina induced accumulation of DNA damage and a sharp increase in apoptosis.

### Blockade of embryonic cell death rescues retinal dysplasia and vision

**Fig. 5.**
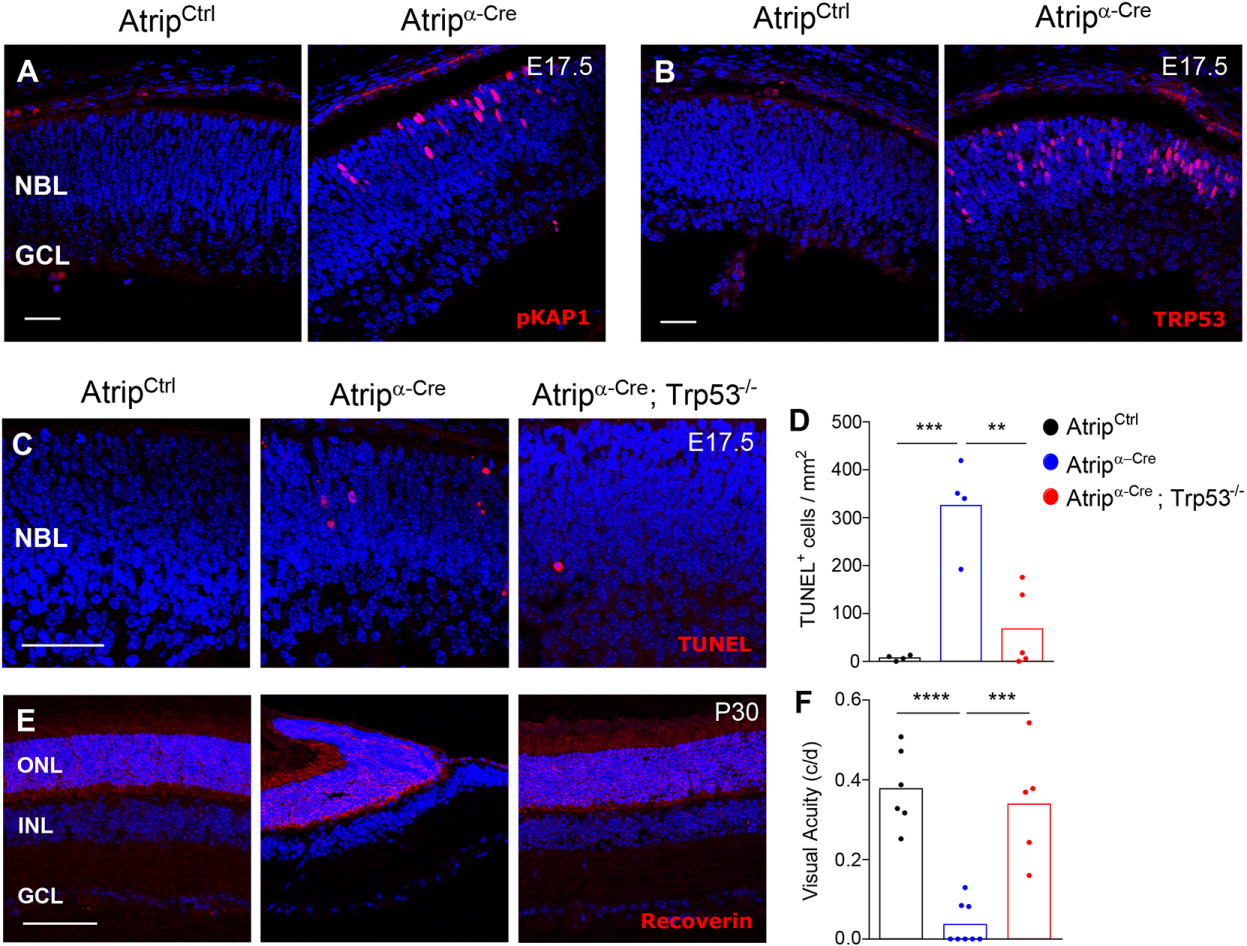
***Trp53* inactivation rescues apoptosis, morphological defects and visual impairment caused by ATRIP loss in RPCs.** (A,B) Representative confocal images of phosphorylated KAP1 (pKAP1, S324) (A) and TRP53 (B) immunostaining in the *Atrip^Ctrl^* and *Atrip^α-Cre^* retinas at E17.5. (C,D) Representative confocal images (C) and quantification (D) of the TUNEL assay in *Atrip^Ctrl^* and *Atrip^α-Cre^* and *Atrip^DKO^* retina at E17.5. (E) Recoverin immunostaining of *Atrip^Ctrl^* and *Atrip^α-Cre^* and *Atrip^DKO^* retinas at P30. (F) Behavioral optomotor response analysis in the *Atrip^Ctrl^* and *Atrip^α-Cre^* and *Atrip^DKO^* mice at P60. Statistical analysis: one-way ANOVA followed by Tukey's post hoc test. **P<0.01; ***P<0.001; ****P<0.0001. Scale bars: 50 µm. c/d, cycles/degree; GCL, ganglion cell layer; INL, inner nuclear layer; NBL, neuroblastic layer; ONL, outer nuclear layer.

Persistent replicative stress can result in DNA double-strand breaks (DSB), ATM kinase activation and TRP53-mediated cell death (Olcina et al., 2013; Reinhardt and Schumacher, 2012; Zeman and Cimprich, 2014). To test whether these molecular mechanisms mediate the apoptosis of the *Atrip*-deficient RPCs, we analyzed ATM activation and TRP53 stabilization. Phosphorylation of the ATM-specific target KAP1 (pKAP1^+^) (Fig. 5A) and higher levels of TRP53 protein (Fig. 5B) in the *Atrip^α-Cre^* retinas at E17.5 suggested that TRP53 mediates the apoptosis of the *Atrip*-deficient RPCs.

**Fig. 6.**
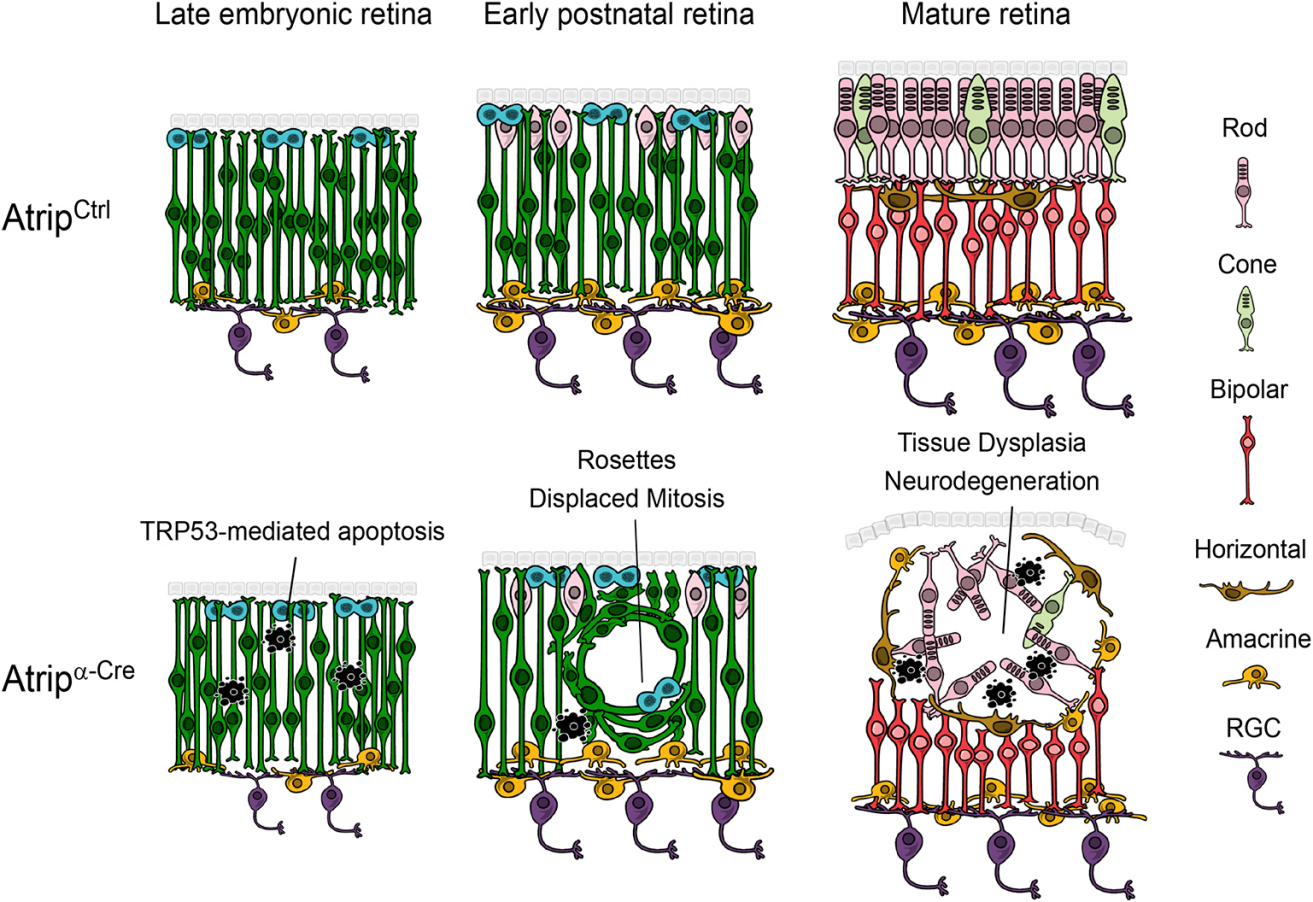
**Progenitor cell death drives retinal dysplasia and neuronal degeneration in a mouse model of ATRIP-Seckel syndrome.** We propose a model in which ATRIP plays a role in regulating the replicative stress response in retinal progenitor cells (RPCs, dark green). Its loss leads to *Trp53*-mediated apoptosis (black pyknotic nuclei) during embryogenesis. We speculate that the ∼50-fold increase in the apoptosis of RPCs might interfere with the movements of progenitor cells and newborn neurons, driving lamination defects and retinal malformations during postnatal development and subsequent neurodegeneration. RGC, retinal ganglion cell.

In neural progenitor cells of the brain, TRP53 mediates apoptosis induced by ATR loss (Lang et al., 2016; Lee et al., 2012). To test whether TRP53 drives the apoptosis of the *Atrip*-deficient RPCs, we generated *Atrip^α-Cre^*; *Trp53*^−/−^ mice. TUNEL assays revealed that *Trp53* inactivation in the *Atrip*-deficient retinas rescued the apoptosis caused by ATRIP loss (Fig. 5C,D), confirming that TRP53 is also required for the replicative stress-induced apoptosis in the developing retina. Although TRP53 drives the apoptosis in both neural tissues, its inactivation does not prevent adult brain malformations caused by ATR loss (Lang et al., 2016; Lee et al., 2012). Surprisingly, the *Atrip/Trp53* double-knockout (DKO) adult retinas bore a phenotypic resemblance to control retinas, displaying normal morphology, lamination and an intact ONL (Fig. 5E). In addition, the *Atrip/Trp53* DKO *(Atrip^DKO^*) mice displayed a normal optomotor response (Fig. 5F). Altogether, these findings indicate that, unlike other neural tissues, the TRP53-mediated apoptosis of the *Atrip*-deficient RPCs during embryogenesis is the key event that leads to defective morphogenesis, subsequent photoreceptor degeneration and loss of vision (Fig. 6).

## DISCUSSION

Seckel syndrome is genetically heterogeneous, and mutations in DDR genes (*ATRIP* and *ATR*), DNA repair factors (*NSMCE2*, *DNA2*, *TRAIP* and *CtIP*) and components of the centrosome (*NIN*, *CEP63*, *CEP152* and *CENPJ*) have been reported (Khetarpal et al., 2016). The biological basis of the developmental defects associated with the loss of *ATR* and other Seckel-causing genes has been studied previously (Gabriel et al., 2016; Lee et al., 2012; McIntyre et al., 2012; Murga et al., 2009). In a seminal study, Murga and colleagues referred to the intrauterine programming model (Barker, 1995; Fowden et al., 2006) to explain the premature aging phenotypes caused by an ATR-Seckel mutation in mice (Murga et al., 2009). In this model, the deregulated RSR caused by loss of ATR function in embryonic progenitor cells would be the underlying cause of the progeroid manifestations (Murga et al., 2009).

Our findings on retinal development and degeneration are consistent with this model, because they reinforce that unregulated replicative stress during embryogenesis causes developmental defects that lead to severe functional deficits in adulthood. In particular, our results reveal a causal relationship between a cellular mechanism (progenitor cell death caused by defective RSR) and a CNS dysfunction (loss of vision), elucidating the neurodevelopmental defects in a model of ATRIP-Seckel syndrome. Overall, our results demonstrate that ATRIP is crucial for retinal morphogenesis and identify the apoptosis of the RPCs as a driver of retinal malformations and neurodegeneration following deregulated RSR. Our findings are summarized in Fig. 6.

Deletion of *Atr* in postmitotic neurons of the adult brain had no effect on neurological function (Ruzankina et al., 2007). In contrast, mice harboring a hypomorphic mutation of *Atr* exhibited postnatal degeneration of postmitotic photoreceptor neurons and visual impairment (Valdés-Sánchez et al., 2013). Detection of ATR protein at the connecting cilia and reduced ciliary length in these *Atr*-deficient retinas led to the suggestion of a replicative stress-independent function role of ATR in photoreceptor cilia (Valdés-Sánchez et al., 2013). In addition, alterations in the morphology and function of cilia were described in *Atr*-deficient cell lines and in *Atr*-depleted zebrafish (Stiff et al., 2016). Here, we demonstrate that ATRIP is not essential for the survival and function of postmitotic photoreceptor neurons, as shown by conditional inactivation of *Atrip* in rod photoreceptors. Thus, although an RSR-independent role of ATR in cilia might contribute to the lack of photoreceptor function in ATR-Seckel mice, our findings show that the degeneration caused by ATRIP loss is not attributable to cell-autonomous functions in photoreceptors. To our knowledge, no study has explored whether ATRIP deficiency results in dysfunction of cilia. Further research is required to determine whether the potential roles of ATR in the cilia of photoreceptors depend on its interaction with ATRIP.

TRP53-mediated cell-cycle arrest and apoptosis are common cellular responses to defective RSR and DSB (Hafner et al., 2019). In both developing organs and adult organs, loss of the ATRIP-ATR complex in progenitor cells activates DDR, affecting progenitor cell survival and/or proliferation (Lang et al., 2016; Lee et al., 2012; Matos-Rodrigues et al., 2020 preprint; Murga et al., 2009; Ruzankina et al., 2007). It has been shown that the ATR-Seckel mice have sustained replicative stress that results in TRP53 accumulation and neural progenitor cells apoptosis (Murga et al., 2009). Moreover, combined loss of ATR and TRP53 revealed the striking phenotypes of synthetic lethality, a finding that was crucial for the development of current approaches targeting ATR in cancer therapies (Lecona and Fernandez-Capetillo, 2018; Murga et al., 2009; Ruzankina et al., 2009). Similar to ATR-Seckel mice, CNS-specific *Atr* inactivation reinforced that ATR loss affected the survival of progenitor cells in the embryonic brain. Interestingly, although these *Atr-*deficient neural progenitor cells accumulate TRP53 and undergo apoptosis, inactivation of *Trp53* in *Atr*-deficient brains did not rescue brain structure and function (Lang et al., 2016; Lee et al., 2012). Therefore, the cause of CNS developmental malformations following defective RSR has remained unclear. Here, we have shown that inactivation of *Atrip* in the RPCs during embryogenesis led to the accumulation of DNA damage, ATM activation and TRP53-mediated apoptosis, without a major impact on RPC proliferation and neurogenesis. In contrast to previous models of ATR-ATRIP loss of function, in *Atrip*-deficient retinas, the blockade of apoptosis rescued tissue morphogenesis and function completely, suggesting that the apoptosis of the RPCs is the major driver of retinal malformations. These results demonstrate that the defective RSR has variable impacts on different progenitor cells of the developing CNS (Lee et al., 2012; Li et al., 2012; Rodrigues et al., 2013). Evaluation of the physiological levels of endogenous replicative stress in different types of neural progenitors might help elucidate the tissue-specific effects of DDR mutations in congenital malformations. Furthermore, understanding why retinal progenitor cells display a distinct epistatic relation between ATRIP-ATR and TRP53 is an exciting question for future investigations that might have therapeutic relevance.

Naturally occurring apoptosis is crucial for tissue morphogenesis (Fuchs and Steller, 2011); however, excessive cell death can cause a variety of developmental disorders, including microcephaly and neural tube defects (Garcez et al., 2016; Little and Dwyer, 2019; Pani et al., 2002). More specifically, regarding the DNA damage response during development, it was previously shown that human embryos exposed to high doses of radiation present with retinal rosettes (Goldstein and Wexler, 1931). Apoptosis induced by replicative stress was also associated with defective morphogenesis and tissue dysplasia (Muñoz et al., 2017). These findings suggested a possible link between replicative stress and retinal malformations. Here, we show that replicative stress-induced apoptosis of progenitor cells during retinogenesis might have a causative role in structural malformations of the retina.

This discovery raises the question of how the death of RPCs during embryogenesis could disrupt retinal structure. Dying cells can modify the mechanical properties of the tissue and affect the movements of the surrounding cells (Ambrosini et al., 2017; Pérez-Garijo and Steller, 2015). Thus, given that cell migration is key for the formation of retinal architecture (Amini et al., 2017), we speculate that the ∼50-fold increase in the apoptosis of RPCs upon ATRIP loss might interfere with the movements of progenitor cells and newborn neurons, affecting tissue integrity. Misplaced neurons could exacerbate the disorganization by preventing later-born neurons from reaching their destination (Icha et al., 2016). Ultimately, lamination defects could lead to the degeneration of misplaced neurons because of the lack of support from adjacent tissues, in particular owing to the interdependence between photoreceptor neurons and the adjacent retinal pigmented epithelium (Boulton and Dayhaw-Barker, 2001; Kim et al., 2013). Therefore, although the death of newly born retinal neurons cannot be excluded, we propose that RPC apoptosis throughout retinogenesis might have ripple effects that cause the extensive neuronal degeneration and loss of vision observed in our ATRIP-Seckel model. It is possible that similar non-cell-autonomous effects of apoptosis might also contribute to the malformations of the neural tube and other neuroepithelial tissues (Alcantara and O'Driscoll, 2014; Ikeda et al., 2001; Nikolopoulou et al., 2017).

To our knowledge, no morphological analyses (e.g. optical coherence tomography or tissue histology) of the retinas of ATR/ATRIP-Seckel patients have been performed. Therefore, follow-up studies using primary tissue and retinal organoids from patient-derived induced pluripotent stem cells will be important to confirm the contribution of apoptosis and lamination defects to the etiology of the retinal malformations associated with Seckel microcephalic primordial dwarfism syndromes.

## MATERIALS AND METHODS

### Ethics statement and mice

Experimental procedures with animals were approved by the Committee of Ethics in Animal Use (CEUA) of the Health Science Center (CCS) in Brazil and approved by the governmental review board of the state of Baden-Württemberg (Regierungspräsidium Karlsruhe-Abteilung 3-Landwirtschaft, Ländlicher Raum, Veterinär-und Lebensmittelwesen) in Germany. All aspects of the mouse work were carried out following strict guidelines to ensure careful, consistent and ethical handling of the mice.

Transgenic mice lines used in this work were as follows: α-Cre [Tg(Pax6-cre,GFP)^2Pgr^] (Marquardt et al., 2001), Trp53 null (C3Ou.129S2 B6-TrTrp53^tm1Tyj/J^) (Lowe et al., 1993), iRho-Cre (Li et al., 2005) and Atrip Floxed (B6; Sv129-*Atrip*^tm1.1pof^) (Matos-Rodrigues et al., 2020 preprint). The mice were identified as follows: *α*-Cre^+/−^; Atrip^Flox/Flox^=*Atrip^α-Cre^*; *α*-Cre^+/−^; Atrip^Flox/Flox^; Trp53^−/−^=*Atrip^DKO^*; iRho-Cre^+/−^; Atrip^Flox/Flox^=*Atrip^iRho-Cre^*. Mice harboring wild-type *Atrip* alleles with or without *α*-Cre or iRho-Cre transgenes, or Atrip^Flox/Flox^ without Cre transgenes, were identified as *Atrip^Ctrl^*.

### RNA extraction, complementary DNA synthesis and real-time RT-PCR

Retinas from three different mice of the same litter were dissected in cold PBS and lysed in 1 ml of Trizol (Life Technologies/Thermo Fisher Scientific, 15596026). After mechanical lysis of the tissue using a 100 U syringe, standard Trizol extraction was performed and the pellet resuspended in 20 μl ultrapure water (Life Technologies/Thermo Fisher Scientific, 10977). RNA concentration and purity were determined using a Nanodrop spectrophotometer (Eppendorf). Analysis of ribosomal RNA integrity was performed by electrophoresis in 1% agarose (Sigma-Aldrich, A9539) gels. After DNase treatment (rDNase kit, Ambion, AM1906), following the manufacturer's instructions, contamination with genomic DNA was verified by PCR using primers for genomic DNA, followed by electrophoresis in agarose gel. Complementary DNA (cDNA) was synthetized using the First-strand cDNA synthesis kit (GE Healthcare, 27-9261-01), following the manufacturer's instructions.

Primers for real-time RT-PCR were designed using the following criteria: amplicon size, between 80 and 200 bp; melting temperature of 60°C; and 50% of GC (G, guanine; C, cytosine). A melting curve and RT-PCR product electrophoresis in agarose gel were performed to verify the presence of a single amplicon of the expected size. Standard curves using serial dilutions of a cDNA mix from the lens and retina of different developmental stages were performed to analyze linearity and efficiency. Linearity was ascertained using the coefficient of determination (*r*²) and primer efficiency (*E*) by the equation *E*=10(−1/slope) (Pfaffl, 2001).

Real-time RT-PCRs were performed in 96-well optic plates (Applied Biosystems, N801-0560) in an Applied Biosystems ABI7500 thermocycler. The primers used for real-time RT-PCR were as follows: *Atr* forward 5′-TGCTATTCAGGAGTTGCTTTCT-3′ and reverse 5′-GGACATGCTCAGGGAATCTTT-3′; *Atrip* forward 5′-TCTCCAGAAAGCTCCAATCAC-3′ and reverse 5′-TCTCCAGAAAGCTCCAATCAC-3′. Taqman primers and probes were as follows: *Gpi1* forward 5′-TCCGTGTCCCTTCTCACCAT-3′, reverse 5′-GGCAGTTCCAGACCAGCTTCT-3′ and probe 5′-CTCCCTGCCCAGAGCGCACC-3′; and β-actin forward 5′-AGCCACCCCCACTCCTAAGA-3′, reverse 5′-TAATTTACACAGAAGCAATGCTGTCA-3′ and probe 5′- ATGGTCGCGTCCATGCCCTGA-3′. For SYBR Green (Applied Biosystems, 4367659), reactions had 12.5 μl of SYBR Green 2× mix, 2 μl of diluted cDNA (1:10), 0.5 μl (5 μM) of each primer and 9.5 μl of UltraPure water (Gibco, 10977). For Taqman (Life Technologies/Thermo Fisher Scientific, 4369016), reactions had 10 μl of Taqman 2×, 1 μl of diluted cDNA (1:10), 0.4 μl (5 μM) of each primer, 0.2 μl of probe (5 μM) and 8 μl of UltraPure water. The cycling conditions were as follows: 50°C for 2 min, 95°C for 10 min and 40 cycles of 95°C for 15 s and 60°C for 60 s. Each sample was reacted in duplicate, and only duplicates with <0.5 *Ct* variation were analyzed further. The comparative method for relative quantification (2^−ΔΔ*Ct*^) was applied to determine the relative quantity of a target compared with the mean of the two reference genes (*Gpi1* and β-actin).

### Measurement of the volume of the eye

The volume of postnatal and adult eyes was measured as previously described (Cavalheiro et al., 2014).

### Immunostaining and TUNEL assay

Slides were washed with PBS and antigen retrieval was applied using a citrate buffer (pH 6). The following antibodies and dilutions were used: anti-Ser10 pH3 (1:200, Cell Signaling Technology, cat# 9701), anti-active caspase-3 (1:100, BD Biosciences, cat# 559565), anti-γH2AX (1:300, Millipore, cat# 05-636), anti-γH2AX (1:200, Abcam, cat# ab11174), anti-BrDU (1:3, General Electric, cat# RPN20), anti-Sox9 (1:100, Abcam, cat# ab5535), anti-TRP53 (CM5) (1:300, Vector Laboratories, cat# VP-P956), anti-β-catenin (1:100, BD Bioscience, cat# 610153), anti-syntaxin (1:100, Sigma-Aldrich, cat# S0664), anti-Prox1 (1:100, Covance, cat# PRB-238C), anti-PKCa (1:200, Millipore, cat# 05-154), anti-Opsin-LM (1:100, Millipore, cat# AB5405), anti-rhodopsin (1:200, Millipore, cat# MABN15), anti-Brn3a (1:300, Santa-Cruz Biotechnology, cat# SC31989), anti-pKAP1 (S384) (1:200, Bethyl Laboratories, cat# A300767A), anti-laminin 1+2 (1:200, Abcam, cat# ab7463), anti-recoverin (1:5000, Millipore, cat# AB5585), anti-calretinin (1:100, Millipore, cat# MAB1568).

Immunofluorescence reactions were developed by different methods: biotin-conjugated secondary antibody followed by ABC complex and Cy3-tyramide kit (∼555 nm excitation, red staining; Perkin Elmer, cat# FP1046) or an Alexa secondary antibody (∼488 nm excitation, green staining; 1:500, Life, cat# A11001). Fluorescent nuclear counterstaining was performed using 4′,6-diamidino-2-phenylindole (DAPI; Lonza, cat# PA3013).

To label S-phase cells *in vivo*, intraperitoneal injections of 50 μg/g of body weight of BrDU (Sigma-Aldrich, cat# B5002) were performed. Eyes were collected 1 h after injection. TUNEL (Promega, cat# G7362) analysis was performed following the manufacturer's instructions. Fluorescent images were captured using a Leica TCS-SP5 with an AOBS system or a Zeiss Axiovert fluorescence microscope.

### Western blotting

Protein extraction and SDS-PAGE protein separation were performed as previously reported (Rocha-Martins et al., 2012). The protein concentration was determined using the Bradford method. An input of 10 µg of lysate was separated using SDS-PAGE gels and then transferred to nitrocellulose membranes. For immunoblotting, membranes were blocked for 1 h in 5% milk Tris buffered saline-Tween 0.5% (TBS-T) solution at room temperature before incubation with primary antibodies. The incubation with primary antibodies was performed overnight at 4°C: ATR (1:500, Santa Cruz Biotechnology, cat# SC1887) and α-tubulin (1:10000, Santa Cruz, cat# SC32293). Horseradish peroxidase-conjugated secondary antibodies (1:1000) were incubated for 1 h at 4°C (Cell Signaling Technology, anti-mouse IgG, cat# 7076; anti-rabbit IgG, cat# 7074). The ECL system (cat# RPN2132) was used according to the manufacturer's instructions, and chemiluminescence was captured using ChemiDoc MP (BioRad) equipment.

### Optomotor response test

Measurements of visual acuity by optomotor response were performed using OptoMotry as previously described (Cavalheiro et al., 2017; Rocha-Martins et al., 2019). Experimenters were blind in relation to mice genotypes, and the visual accuracy threshold was determined by systematic increments of the spatial frequency until the animal no longer responded.

### Experimental design, quantifications and statistical analysis

At least three mice were used in each group per analysis, and the number of mice used in each experiment was plotted as a dot in each graph (black dots for *Atrip^Ctrl^*; blue dots for *Atrip^α-Cre^*; red dots for *Atrip^DKO^* mice). For every statistical analysis, the number of mice per experiment is represented in each graph or legend. Quantifications of the histological sections were performed exclusively on the retinal periphery of at least three biological sections from each biological sample. GraphPad Prism software was used for statistical analysis. Student's *t*-test or one-way ANOVA was performed as indicated in each figure legend. Computations assumed the same scatter (s.d.) and Gaussian distribution between groups. *P*-values were based on two-sided tests. Standard methods (http://powerandsamplesize.com/) were applied to determine the power to detect a prespecified effect size. Mice were allocated to experimental groups in accordance with their genotypes. The experimenters were blind only in relation to the genotype of mice in each experimental group for the optomotor response test.

## Supplementary Material

Supplementary information
