# Supplementary material for: Progenitor death drives retinal dysplasia and neuronal degeneration in a mouse model of ATRIP-Seckel syndrome
Source: Dis Model Mech. 2020 Oct 30;13(10):dmm045807. doi: 10.1242/dmm.045807 (PMC7648607; doi:10.1242/dmm.045807)
Supplement: Supplementary information [file dmm-13-045807-s1.pdf]

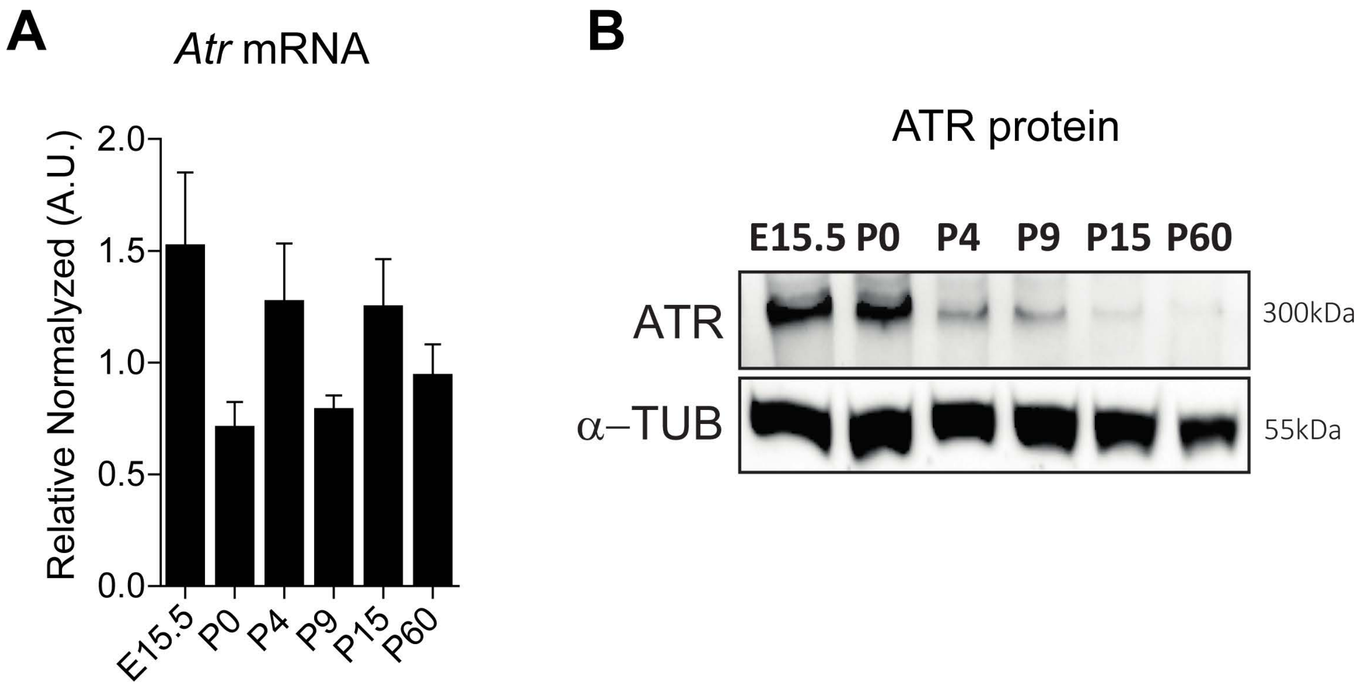

Fig. S1. *Atr* expression pattern during retinal development. (A) Real-time RT-PCR for *Atr*. TaqMan probes for *Gpi1* and *Actb* were used as loading controls (n=3). **(B)** Western blot analysis of the ATR protein content in the E15.5, P0, P4, P9, P15 and P60 mouse retinas. Various commercial antibodies were tested (NB100-359, Bioworld BS1510, LSBIO C117796, ATRIP F-7 sc-365383, ThermoFisher PA1-519 and Cell signaling technologies #2737) to examine endogenous levels of ATRIP protein during retinogenesis by western blot. Either endogenous mouse ATRIP protein was not detectable or the detected ~85kD band was unspecific.

Disease Models & Mechanisms • Supplementary information

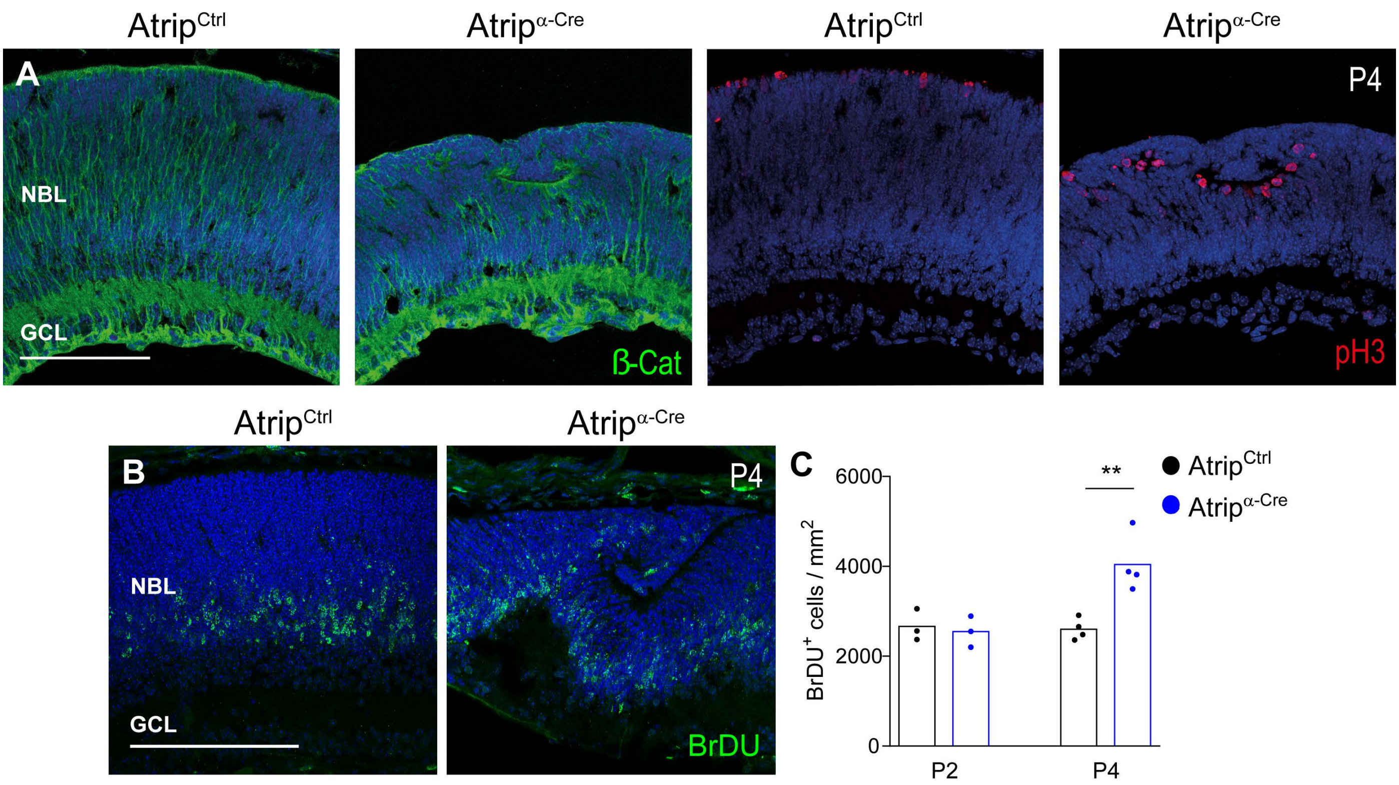

Fig. S2. Inactivation of *Atrip* leads to postnatal apical-basal polarity defects and proliferation defects. (A) Representative confocal images of the *Atrip*^{Ctrl} and *Atrip*$^{\alpha\text{-}Cre}$ retinas double stained for β-catenin (β-cat) and pH3 at P4. **(B)** Representative images and quantitative analysis of BrDU positive cells per mm² at P2 and P4 in the *Atrip*^{Ctrl} and *Atrip*$^{\alpha\text{-}Cre}$ retinas. Scale bar: 100 µm. NBL: Neuroblastic layer.

Disease Models & Mechanisms • Supplementary information

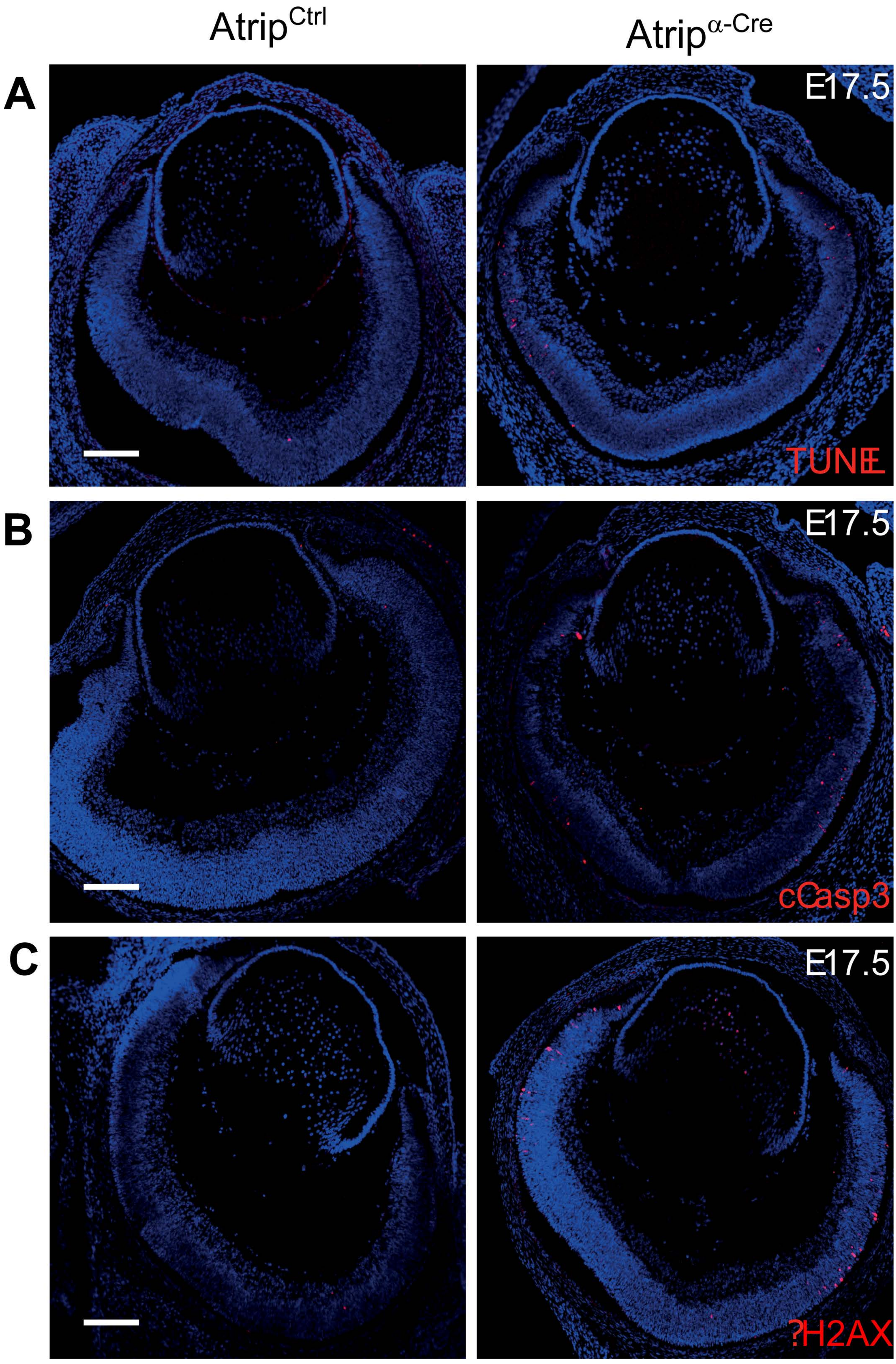

Disease Models & Mechanisms • Supplementary information

Fig. S3. ***Atrip***$^{\alpha\text{-}Cre}$ **retinas show apoptotic cell death and DNA damage in the retinal periphery. (A-C)** TUNEL assay (**A**), cCasp3 immunofluorescence (**B**) and γH2AX immunofluorescence (**C**) representative confocal images from eye cryosections in *Atrip*^{Ctrl} and *Atrip*$^{\alpha\text{-}Cre}$ samples at E17.5. Scale bar: 100µm.
